# Previous Gestational Diabetes Mellitus and Markers of Cardiovascular Risk

**DOI:** 10.1155/2012/458610

**Published:** 2012-03-18

**Authors:** Nikolaos Vrachnis, Areti Augoulea, Zoe Iliodromiti, Irene Lambrinoudaki, Stavros Sifakis, George Creatsas

**Affiliations:** ^1^2nd Department of Obstetrics and Gynecology, University of Athens Medical School, Aretaieio Hospital, 11526 Athens, Greece; ^2^Obstetric and Gynecological Unit and Research Center, Evgenideio Hospital, University of Athens, 11526 Athens, Greece; ^3^Department of Obstetrics and Gynaecology, University Hospital of Heraklion, 71110 Heraklion, Crete, Greece

## Abstract

The prevalence of gestational diabetes mellitus (GDM) in the developed world has increased at an alarming rate over the last few decades. GDM has been shown to be associated with postpartum diabetes, insulin resistance, hypertension, and dyslipidemia. A history of previous GDM (pGDM), associated or not with any of these metabolic abnormalities, can increase the risk of developing not only type 2 diabetes mellitus but also cardiovascular disease (CVD) independent of a diagnosis of type 2 diabetes later in life. In this paper we discuss the relationship among inflammatory markers, metabolic abnormalities, and vascular dysfunction in women with pGDM. We also review the current knowledge on metabolic modifications occurring in normal pregnancy and the link between alterations of a normal metabolic state with the long-term maternal complications that may result in increased CVD risk. Our review of studies on pGDM prompts us to recommend that these women be considered a population at risk for later CVD events, which however could be avoided via the use of specially designed follow-up programs in the future.

## 1. Introduction

Gestational diabetes mellitus (GDM) is any degree of glucose intolerance with onset or first recognition during pregnancy [1, 2]. In early gestation fasting blood glucose is lower and insulin sensitivity decreases slightly. This is followed by progressively increasing insulin resistance in the second and third trimesters with a borderline increase of insulin production or hyperinsulinemia. Furthermore, insulin resistance occurs as a result of placental hormones that antagonize insulin, estrogen, progesterone, human placental lactogen (HPL), human placental growth hormone, cortisol, prolactin, and tumor necrosis factor-alpha (TNF-*α*) [3]. The above different pathophysiologic mechanisms accompanying pregnancy result in metabolic changes that allow for higher postprandial maternal glucose. Pregnancy is a hyperinsulinemic state which may develop into impaired glucose tolerance if insulin secretion is unable to compensate for pregnancy-associated insulin resistance [3–5].

The condition of GDM is a state of chronic low-grade subclinical inflammation characterized by abnormal production of cytokine and mediators and activation of a network of inflammatory signaling pathways. Although the characteristic of GDM is insulin resistance, the exact mechanism involved in this process is still unknown. The increased insulin resistance during pregnancy has been, as just described, attributed to cortisol and gestational hormones, but more recent data have shown that cytokines may also be involved in this process [6]. The most significant maternal risk is that of development of metabolic syndrome characterized by central obesity, dyslipidemia, and insulin resistance, which predispose to increased risk for coronary artery disease, stroke, and type 2 diabetes later in life [7–11].

The incidence of type 2 diabetes in women with previous GDM (pGDM) who were examined six weeks to 28 years postpartum was estimated to range from 2.6% to 70% [12, 13]. Other researchers found that women with pGDM have a 18–50% risk of developing type 2 diabetes mellitus within 5 years following pregnancy [14–17], and diabetes is an established risk factor for CVD [18, 19]. In addition, women with a history of GDM are at increased risk of other cardiovascular risk factors, such as obesity, hypertension, dyslipidemia, and subclinical atherosclerosis [20–22]. It is unclear whether women with a history of GDM who do not subsequently develop type 2 diabetes mellitus are also at an increased CVD risk in the future. The metabolic abnormalities which accompany GDM preceding type 2 diabetes and which remain in effect during the natural course of the disease place women at high risk for CVD [23].

In this paper we review the interrelationship among inflammatory markers, metabolic abnormalities, and endothelium dysfunction in pGDM and discuss whether these women could be considered at risk for cardiovascular disease later in life. Based on the small amount of existing literature, we discuss the inflammatory and metabolic abnormalities underlying the status of pGDM and the potential that endothelial dysfunction is a marker of future CVD risk. To our knowledge, this is the first paper presented in the literature dealing with markers of CVD risk in women with a history of gestational diabetes.

## 2. Surrogate Markers of Increased Cardiovascular Risk

Although the majority of women with GDM return to normal glucose tolerance after delivery, they remain, as a group, at substantially increased risk of developing type 2 diabetes in later life, a known condition that leads to an increased risk for CVD [24].

Inflammation may contribute to atherosclerosis by a variety of mechanisms depending on the stage of the disease. Circulating markers of systemic inflammation have been shown to predict future CVD [25]. These markers include C-reactive protein (CRP), proinflammatory cytokines such as interleukin-6 (IL-6), and soluble adhesion molecules. Most attention has been focused on CRP which, along with IL-6, has been revealed in large prospective studies to be a consistent predictor of future cardiovascular events [26, 27].

**Figure 1:**
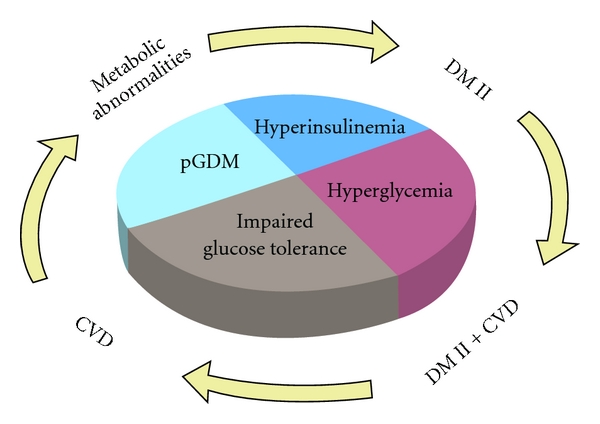
Metabolic status during pregnancy and pGDM may result in outcomes later in life like metabolic abnormalities, DMII, CVD, and DMII + CVD as it is shown following the arrows.

Epidemiological and experimental studies have established the association of markers of subclinical inflammation with CVD, type 2 diabetes, and metabolic syndrome (Figure 1). Pregnancy is a hyperinsulinemic state in which the increased insulin resistance during pregnancy may be attributed not only to gestational hormones but also possibly to cytokines, which, as mentioned in Section 1, may play a role [28–30]. Increased levels of inflammatory markers such as CRP, plasminogen activator inhibitor-1 (PAI-1), and IL-6 are predictors of future establishment of type 2 diabetes and CVD [31–34]. Adiponectin, a peptide with anti-inflammatory properties, has in some studies been associated with a decreased risk of type 2 diabetes and CVD [35, 36].

Markers of endothelial dysfunction, like circulating levels of E-selectin, vascular adhesion molecule-1 (VCAM-1), intercellular adhesion molecule 1 (ICAM-1), as well as inflammatory parameters like CRP and IL-6, have been reported to be associated with CVD in several studies [37–42]. Furthermore, the adhesion molecules E-selectin, VCAM-1, and ICAM-1 are thought to play a major role in the pathogenesis of vascular disease [6]. These molecules are markers of endothelial dysfunction and are expressed on the endothelial wall in response to inflammatory mediators. They contribute to the formation of atherosclerotic plaques and can be detected in soluble form in the circulation [43]. More reliable, and thus of great interest, are the measurements of carotid intima media thickness (IMT) and flow-mediated vasodilatation (FMD). Both indexes have been used in epidemiological studies as surrogate markers of early atherosclerosis. Carotid IMT increases with age, is correlated with cardiovascular risk factors, and identifies subjects at increased risk of severe coronary artery disease and cardiovascular morbidity [37]. Intima media thickness (IMT) is an ultrasound marker of CVD risk. Heiss et al. in their study found positive relation of this marker to cardiovascular risk factors and CVD risk [44].

Mediators of inflammation may exert pathologic action by inducing vascular dysfunction, thus leading to many of the diverse effects of the insulin resistance condition, like hypertension, dyslipidemia, and impaired fibrinolysis [62]. Insulin resistance has been associated with impaired endothelial function, which interacts with coagulation and hypofibrinolysis [63, 64], while hypofibrinolysis and procoagulant activity are linked with increased risk for cardiovascular events (Table 1). It is of note that raised levels of circulating inhibitors of the fibrinolytic system have been observed in patients with insulin resistance [64]. Plasminogen activator inhibitor-1 (PAI-1) is elevated in a variety of clinical situations that are associated with insulin resistance and cardiovascular disorders [65].

Osteoprotegerin (OPG) is a glycoprotein, a soluble member of the tumor necrosis factor (TNF) receptor superfamily, which inhibits receptor activator of nuclear-factor-*κ*B-ligand (RANKL-) mediated osteoclastic bone resorption [66]. It has been reported to be expressed in the arterial wall [67]. Elevated serum OPG levels have been found to be associated with atherosclerosis [68].

Vessel stiffness measured by arterial tonometry is associated with endothelial dysfunction and increased CVD risk [69, 70].

## 3. Studies in pGDM Women for Identification of the Risk of Cardiovascular Complications

The study of women in the pGDM state serves as a model for the detection of early metabolic abnormalities. Normoglycemic women with pGDM have increased insulin resistance and decreased endothelium-dependent vasodilatation when compared with women who had uncomplicated pregnancies [45, 46]. During the first 3–6 months postpartum, women with pGDM had impaired endothelial function assessed by FMD, this tending to confirm the assumption that glucose metabolism derangement is closely related to vascular dysfunction [46]. A cross-sectional study showed that 2–4 years after the postpartum period, pGDM had impaired acetylcholine-induced skin vasodilatation in hand and foot, as assessed by laser Doppler flow, when compared with normal controls [47]. Two cohort studies have reported signs of vascular endothelial dysfunction in vitro and in vivo during pregnancies complicated by GDM. The first study evaluated vascular endothelial function in small subcutaneous arteries dissected from biopsies obtained at cesarean section using vessel myograph and the second during pregnancy with impaired glucose tolerance and gestational diabetes mellitus assessing brachial artery FMD [48, 49].

Heitritter et al. compared biochemical and hemodynamic surrogate markers of CVD in nondiabetic women with and without a history of GDM who were at least one year postpartum and concluded that nondiabetic women with pGDM have evidence of subclinical inflammation, hypoadiponectinemia, and early vascular dysfunction and may be at increased risk of developing CVD [50]. Lower plasma adiponectin concentrations characterize women with pGDM by contrast to controls, independently of the prevailing insulin sensitivity or the degree of obesity and are associated with subclinical inflammation and atherogenic parameters [51]. Bo et al. showed in their study that pGDM women had higher values of markers of endothelial dysfunction and IMT than controls and an increased future CVD risk; however, few data are available concerning the association between pGDM and inflammation markers of endothelial dysfunction [52]. E-selectin and VCAM-1 concentrations were found to be elevated in a cohort study of women with pGDM one year after delivery [53]. A larger study many months postpartum failed to display the same results [54]. Kautzky-Willer et al. demonstrated that pGDM was characterized by persistently raised levels of E-selectin and VCAM-1 12 weeks after delivery [55]. In a large population-based study, women who had GDM in pregnancy compared with controls were at higher risk of CVD events [56].

Akinci et al. observed that OPG serum levels tended to be elevated in pGDM and moreover reported an association with carotid IMT, thus showing that osteoprotegerin may play a role in the pathogenesis of endothelial dysfunction in these women [57]. Furthermore, a very recent study conducted by the same group concluded that OPG was related to CVD risk factors and metabolic syndrome and may be involved in the development of CVD disorders in pGDM [58]. Farhan et al. recorded elevated PAI-1 levels in pGDM [60].

Another study examined the relationship between glycemia during pregnancy and small artery function 2 years postpartum. In this study subcutaneous arteries from gluteal fat biopsy were examined as to structure, stiffness, and vasoconstrictor response using myography. The results showed that vascular pathology is detectable very early in women at risk of type 2 diabetes [59]. Studying the prevalence of abnormal glucose tolerance and metabolic syndrome in a cohort of pGDM, the results demonstrated disturbed carbohydrate metabolism and a clustering of CVD factors in these women [61, 71].

Surrogate markers of increased cardiovascular risk in population-based studies are commonly used in routine practice. However, though in studies of pGDM markers are used that link this condition with future CVD risk [72], the evidence is as yet inadequate for the markers to be applied in the routine followup of these women. Nevertheless, the aforementioned studies are promising, as several of these biochemical and hemodynamic markers may in future prove to be of great value in follow-up programs, contributing to reducing the risk in pGDM for cardiovascular morbidities later in life.

## 4. Conclusions

It has been shown that women with pGDM are more insulin resistant than women with normal carbohydrate tolerance during their pregnancies. Diabetic complications may be in progress during the phase of insulin resistance in pregnancy even in the absence of hyperglycemia, while furthermore there is evidence that pGDM is associated with postpartum diabetes, insulin resistance, hypertension, and dyslipidemia. A history of pGDM can increase the risk of developing not only type 2 diabetes mellitus, which is a major risk factor for the development of cardiovascular disorders, but also CVD independent of the presence of type 2 diabetes. Also mentioned in this paper is the fact that a number of studies have reported pGDM to be additionally associated with the increased prevalence of metabolic syndrome, an important factor of cardiovascular disorders.

Having reviewed the current literature concerning the relationship between inflammatory markers, metabolic abnormalities, and vascular dysfunction in pGDM, we proceeded to evaluate, for the first time to our knowledge, the sum total of this information for the purpose of seeking to identify women at future risk. We additionally reviewed the current knowledge on normal metabolic modifications that occur in pregnancy and the link between these normal modifications and the ensuing long-term complications in this group of women. Based on the evidence related to pGDM, we suggest that these women be considered at an increased risk for subsequent cardiovascular morbidity. Identifying women at increased risk for developing cardiovascular morbidities and, at a later date, placing them in follow-up programs that will include the use of established selected markers, has the potential to substantially hold back their CVD risk in terms of both, lower incidence and reduced severity of cardiovascular events later in life.

## Figures and Tables

**Table 1:** Markers of increased CVD risk in normoglycemic women with GDM or pGDM.

Authors	Number of subjects	Months/years postpartum	Markers of CVD risk
Kousta et al., 2003 [45]	78	3 years	↑ Insulin resistance ↑ lipidemia
Anastasiou et al., 1998 [46]	68	3–6 months	↓ FMD
Hu et al., 1998 [47]	37	2–4 years	↓ Acetylcholine induced vasodilatation
Knock et al., 1997 [48]	32	During cesarean section	↑ Vascular pathology, vessel myography
Paradisi et al., 2002 [49]	38	During GDM pregnancy	↓ FMD
Heitritter et al., 2005 [50]	48	1 year	↓ Adiponectin
Winzer et al., 2004 [51]	108	3 months	↓ Adiponectin
Bo et al., 2007 [52]	195	6-7 years	↑ E-selectin ↑ ICAM-1 ↑ IMT
Thomaseth et al., 1997 [53]	10	1 year	↑ E-selectin ↑ VCAM-1
Lawrence et al., 2002 [54]	265	recent GDM	≈ E-selectin
Kautzky-Willer et al., 1997 [55]	41	3 months	↑ E-selectin ↑ VCAM-1
Shah et al., 2008 [56]	89.500	p GDM	↑ CVD events
Akinci et al., 2008 [57]	76	p GDM	↑ OPG ↑ IMT
Akinci et al., 2011 [58]	195	3 years	↑ OPG
Banerjee et al., 2011 [59]	29	2 years	↑ Vascular pathology, vessel myography
Farhan et al., 2006 [60]	70	recent GDM	↑ PAI-1
Madarász et al., 2009 [61]	107	4 years	↑ CVD risk factors, disturbed carbohydrate metabolism
